# Elevated numbers of PD-L1 expressing B cells are associated with the development of AIDS-NHL

**DOI:** 10.1038/s41598-019-45479-3

**Published:** 2019-06-28

**Authors:** Marta Epeldegui, David V. Conti, Yu Guo, Wendy Cozen, Manuel L. Penichet, Otoniel Martínez-Maza

**Affiliations:** 1 UCLA AIDS Institute, University of California, Los Angeles, California USA; 2 Jonsson Comprehensive Cancer Center, University of California, Los Angeles, California USA; 3 Department of Obstetrics and Gynecology, David Geffen School of Medicine, University of California, Los Angeles, California USA; 4 Department of Preventive Medicine Keck School of Medicine and Norris Comprehensive Cancer Center, University of Southern California, Los Angeles, California USA; 5 Department of Pathology, Keck School of Medicine and Norris Comprehensive Cancer Center, University of Southern California, Los Angeles, California USA; 6 Division of Surgical Oncology, Department of Surgery, David Geffen School of Medicine, University of California, Los Angeles, California USA; 7 Department of Microbiology, Immunology, and Molecular Genetics, David Geffen School of Medicine, University of California, Los Angeles, California USA; 8 The Molecular Biology Institute, University of California, Los Angeles, California USA; 9 Department of Epidemiology, UCLA Fielding School of Public Health, University of California, Los Angeles, California USA

**Keywords:** Immunology, HIV infections

## Abstract

The risk for non-Hodgkin lymphoma (NHL) is markedly increased in persons living with human immunodeficiency virus (HIV) infection, and remains elevated in those on anti-retroviral therapy (cART). Both the loss of immunoregulation of Epstein-Barr virus (EBV) infected cells, as well as chronic B-cell activation, are believed to contribute to the genesis of AIDS-related NHL (AIDS-NHL). However, the mechanisms that lead to AIDS-NHL have not been completely defined. A subset of B cells that is characterized by the secretion of IL10, as well as the expression of the programmed cell death ligand-1 (PD-L1/CD274), was recently described. These PD-L1^+^ B cells can exert regulatory function, including the dampening of T-cell activation, by interacting with the program cell death protein (PD1) on target cells. The role of PD-L1^+^ B cells in the development of AIDS-NHL has not been explored. We assessed B cell PD-L1 expression on B cells preceding AIDS-NHL diagnosis in a nested case-control study of HIV+ subjects who went on to develop AIDS-NHL, as well as HIV+ subjects who did not, using multi-color flow cytometry. Archival frozen viable PBMC were obtained from the UCLA Multicenter AIDS Cohort Study (MACS). It was seen that the number of CD19^+^CD24^++^CD38^++^and CD19^+^PD-L1^+^cells was significantly elevated in cases 1–4 years prior to AIDS-NHL diagnosis, compared to controls, raising the possibility that these cells may play a role in the etiology of AIDS-NHL. Interestingly, most PD-L1^+^ expression on CD19^+^ cells was seen on CD19^+^CD24^++^CD38^++^ cells. In addition, we showed that HIV can directly induce PD-L1 expression on B cells through interaction of virion-associated CD40L with CD40 on B cells.

## Introduction

AIDS-NHL, comprising Burkitt lymphoma (BL), diffuse large B-cell lymphoma (DLBCL), primary effusion lymphoma, and primary central nervous system lymphoma (PCNSL), are the most common cancers seen in persons living with HIV in the United States and other countries with widespread access to effective combination anti-retroviral therapy (cART)^1^. While, the incidence of AIDS, and AIDS-NHL, has decreased in the cART era, NHL remains a significant clinical problem, causing 23–30% of AIDS-related deaths in countries in which persons living with HIV infection have access to cART^2–4^.

Chronic B cell activation associated with HIV infection, as well as the loss of immunoregulation of Epstein-Barr virus (EBV) infected B cells, are believed to contribute to the development of NHL^5–7^. Epidemiologic evidence points to a potential role for immune stimulatory molecules in the etiology of AIDS-NHL, as elevated levels of several of these molecules (IL6, IL10, BCA1/CXCL13, IP10/CXCL10, MCP1, TARC/CCL17, TNFα, BAFF, IL18, sCD14, sCD163, sCD23, sCD27, sCD30, neopterin, κ and λ immunoglobulin free light chains [FLC]) were observed to precede the development of AIDS-NHL^8–15^. Additionally, *in vitro* studies show that certain cytokines induce the expression and activity of activation-induced cytidine deaminase (AICDA) in B cells^16^. AICDA mediates somatic hypermutation (SHM) and double-strand DNA recombination associated with *IgH* class switch recombination (CSR)^17^. AICDA expression/activity also can result in lymphomagenic lesions by inducing oncogene mutation/translocation^18–22^. We and others have shown that HIV virions can directly activate B cells and induce AICDA expression, via host cell-produced stimulatory molecules that are incorporated into HIV, such as CD40 ligand (CD40L)^23,24^. These studies provide strong evidence that B cell activation precedes and may contribute to the development of AIDS-NHL.

A population of B cells with regulatory function, which have been termed regulatory B cells (Bregs), has been recognized^25^. Bregs are analogous to regulatory T cells, or Tregs, which are T cells that can dampen adaptive immune responses via the secretion of inhibitory cytokines, such as IL10 and TGFβ. Bregs require the interaction of CD40L and CD40, expressed on T and B cells, respectively, to function^25^. This Breg population can dampen T cell function, mainly by producing and secreting IL10 and TGFβ, in a manner analogous to that of Treg cells^25^.

In prior work, we observed that serum IL10 levels are elevated over a period of years (1–5 years) preceding AIDS-NHL diagnosis^9,13,26,27^, as well as after AIDS-NHL diagnosis^28^. Additionally, an IL10 genotype that is associated with enhanced IL10 production was seen to be a risk factor for AIDS-NHL^27^. Others have described the important role of IL10 in the modulation of T-cell function in HIV infection^29^. It recently was shown that HIV+ persons, even those who are on cART, display high levels of IL10 expressing B cells^30^, and Breg cells from HIV+ individuals can suppress CD8 function in an IL10-dependent manner^30^. We showed that exposure of resting B cells from HIV-negative donors to HIV virions containing CD40L led to the secretion of high levels of IL10 by these virion-stimulated cells, in addition to AICDA expression^23^. Therefore, HIV appears to have the potential to induce the generation of Breg cells.

A small (n = 12) study by Siewe *et al*. showed that Breg cell numbers appeared to be elevated prior to AIDS-NHL^31^. It has been previously shown that Toll-like receptor (TLR) activated Breg cells up-regulate the programed death-ligand-1 (PD-L1/CD274)^30^, and additionally, that Breg cells can inhibit CD4^+^ T cells through both IL10 and PD-L1^32^. Therefore, expression of PD-L1 on B cells may provide additional pathway, in addition to IL10, for Breg cells to inhibit T cell function.

Here we show that numbers of both Breg cells (CD19^+^CD24^++^CD38^++^ cells) and CD19^+^PD-L1^+^ cells were elevated in the peripheral circulation of HIV+ subjects prior to AIDS-NHL diagnosis, when compared to the levels seen in HIV+ controls who did not develop AIDS-NHL. Moreover, the majority (~80%) of these CD19^+^PD-L1^+^ cells were Breg cells. The presence of such Breg cells raises the possibility that these cells may inhibit T cell function in a dual fashion, through the actions of both IL10 and PD-L1, thereby contributing to lymphomagenesis. We also show that HIV can diretly induce PD-L1 expression on B cells.

## Results

### Study population

Cases and controls were similar in their distributions by recruitment year, CD4^+^ T cell count, and antiretroviral drug therapy, as expected based on the matched design (Table 1). The majority of controls and cases were non-Hispanic whites (65.5% and 89.0% respectively). Cases and controls were matched by age with a mean age of 33 years for both groups. Cases and controls had relatively high levels of CD4^+^ T cells, with a mean of 464 and 533 CD4^+^ T cells/mm^3^ in controls and cases, respectively. The majority were antiretroviral drug naïve (100% of controls and 95% of cases). All AIDS-NHL cases had systemic lymphoma, equally distributed between DLBCL and BL. The HIV-negative group was 100% male, with a mean age of 38 ± 9 years and mean CD4^+^ T cells/mm^3^ of 975 ± 168. The majority of the HIV-negative controls were white non-Hispanic and Hispanic (66.5% and 28% respectively).

**Table 1:** Select characteristics of the study population.

	HIV-infected controls	AIDS-NHL cases
	n = 18	n = 18
age, median ± SD	33 ± 5	33 ± 4.9
sex		
male	100%	100%
female	0%	0%
CD4, median ± SD	464 ± 216	533 ± 422
median months prior to NHL (visit)	n/a	73.5 ± 26.4
HAART		
yes	0%	5%
no	100%	95%
race		
white, non-Hispanic	66.5%	89.0%
black, non-Hispanic	5.5%	0.0%
Hispanic	28.0%	5.5%
other		5.5%
tumor EBV status		
negative	n/a	33%
positive	n/a	33%
unknown	n/a	33%
tumor subtype		
BL		50%
DLBCL		50%

*Values for each participant at the PBMC sample date > 4 years prior to AIDS-NHL diagnosis.

SD: standard deviation.

### Bregs (CD19^+^CD24^++^CD38^++^) are elevated prior to AIDS-NHL diagnosis

**Figure 1:**
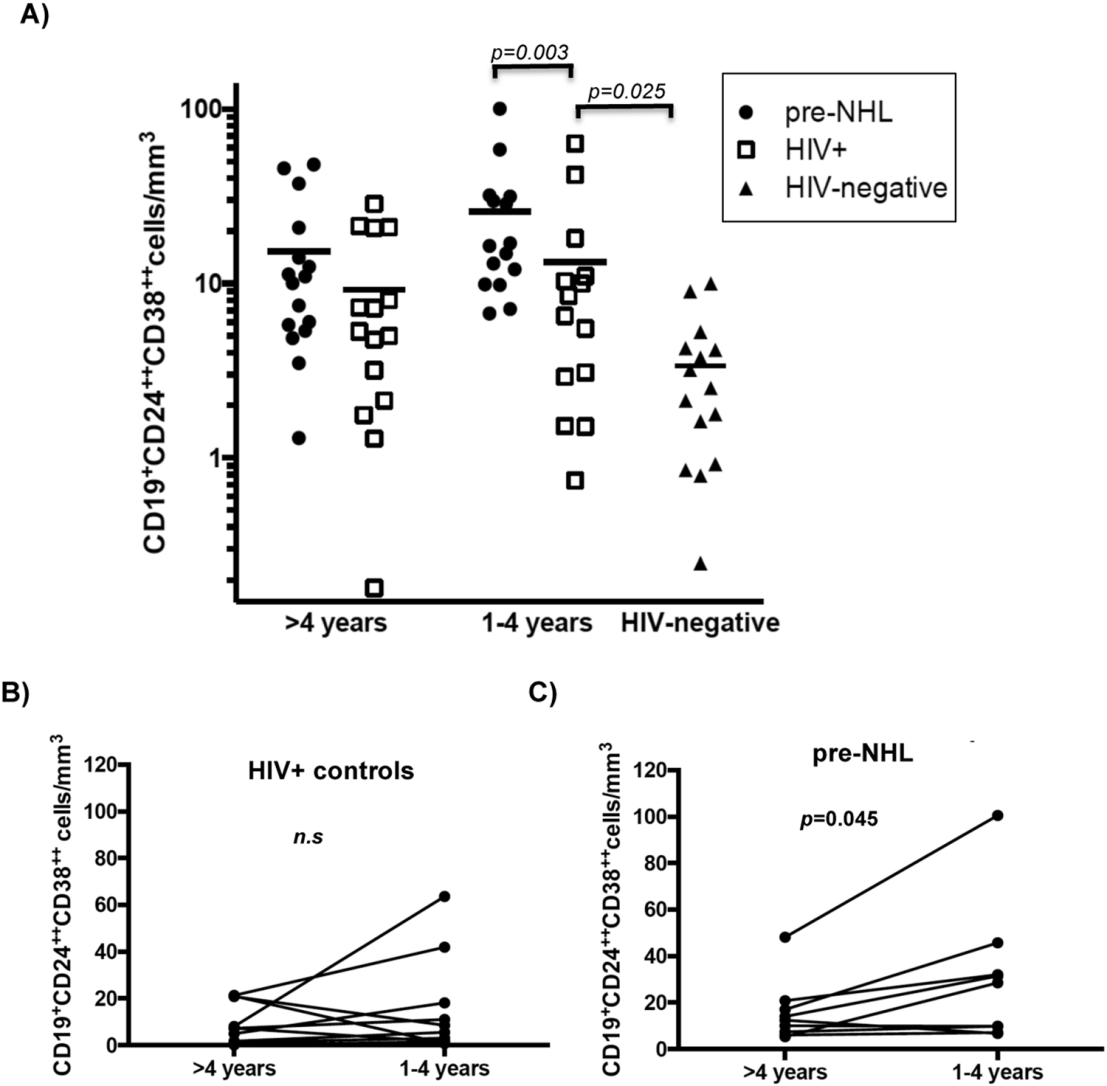
Bregs were elevated prior to AIDS-NHL. (**A**) Multi-color flow cytometry for Breg cell phenotype (CD19^+^CD24^++^CD38^++^) was performed on prospectively collected PBMC from AIDS-NHL cases and matched HIV+ and HIV-negative controls. Breg cells were gated as CD19^+^CD24^++^CD38^++^; absolute numbers of Breg cells (cells/mm^3^) were measured 1–4 years and >4 years prior to AIDS-NHL diagnosis. Lines in represent means. (**B**,**C**) Absolute numbers of Breg cells in PBMC from HIV+ controls are shown at the two different visits; lines represent each individual changes across visits; lines represent each individual changes across visits. *p*-values were calculated using either an F-test or t-test for the difference in log means in a linear mixed model, respectively.

We stained PBMC from HIV+ cases and matched HIV+ controls with antibodies that characterize the Breg cell phenotype (CD19^+^CD24^++^CD38^++^), and assessed these cells using multi-parameter flow cytometry. We observed that PBMC Breg cells were significantly elevated in AIDS-NHL cases 1–4 years prior to their NHL diagnosis (p = 0.003), when compared to their HIV+ matched controls (Fig. 1A). However, Breg cells were not significantly elevated in the circulation of HIV+ NHL cases >4 years prior to diagnosis, when compared to their HIV+ matched controls. PBMC collected from AIDS-NHL cases 1–4 years prior to NHL diagnosis had significantly higher numbers of Breg cells compared to those collected (in the same individuals) > 4 years prior to diagnosis (p = 0.045). However, there was no significant difference in Breg numbers across visits in the HIV+ matched controls (Fig. 1B,C). Lastly, we did observe differences in numbers of Breg cells when we compared the HIV+ and HIV-negative control groups (p = 0.025), suggesting that HIV infection plays a role in increased numbers of Breg cells (Fig. 1A); however, individuals who went on to develop AIDS-NHL displayed even higher levels of Breg cells.

### CD19^+^PD-L1^+^ are elevated prior to AIDS-NHL diagnosis

**Figure 2:**
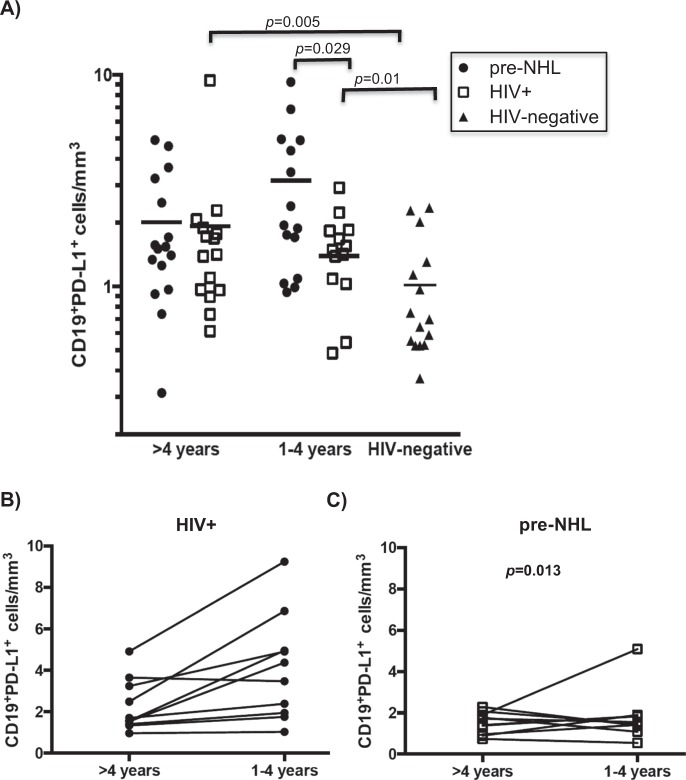
CD19^+^PD-L1^+^ cells were elevated prior to AIDS-NHL diagnosis. (**A**) Multi-color flow cytometry was performed in prospectively collected PBMC from AIDS-NHL cases, as well as from matched HIV+ controls who did not develop NHL and from HIV-negative controls. Absolute numbers of CD19^+^PD-L1^+^ (cells/mm^3^) were measured 1–4 years before AIDS-NHL and >4 years prior to AIDS-NHL. (**B**,**C**) Absolute numbers of CD19^+^PD-L1^+^ cells in PBMC from HIV+ controls are shown at the two different visits; lines represent each individual changes across visits; lines represent each individual changes across. *p*-values were calculated using either an F-test or t-test for the difference in log means in a linear mixed model, respectively.

Recent reports indicate that B cells that express PD-L1 on their surface can impair/inhibit T cell function^32^. To define PD-L1 expression on B cells, we stained PBMC with anti-CD19 and anti-PD-L1, and assessed these cells by flow cytometry, finding a significant increase in the number of PD-L1^+^ B cells in the circulation of AIDS-NHL cases 1–4 years, but not >4 years, prior to NHL diagnosis (p = 0.029) (Fig. 2A). PD-L1 expression on B cells was elevated in all HIV+ subjects, regardless of case status, compared to HIV-negative controls (p = 0.005 and p = 0.01), suggesting that HIV infection is associated with PD-L1 expression on B cells (Fig. 2A). Moreover, we observed that the levels of PD-L1^+^ B cells increased closer to AIDS-NHL diagnosis, compared to the levels of these cells detected at an earlier time (p = 0.013) (Fig. 2B,C).

### Breg cells (CD19^+^CD24^++^CD38^++^) were more elevated in those who developed DLBCL

**Figure 3:**
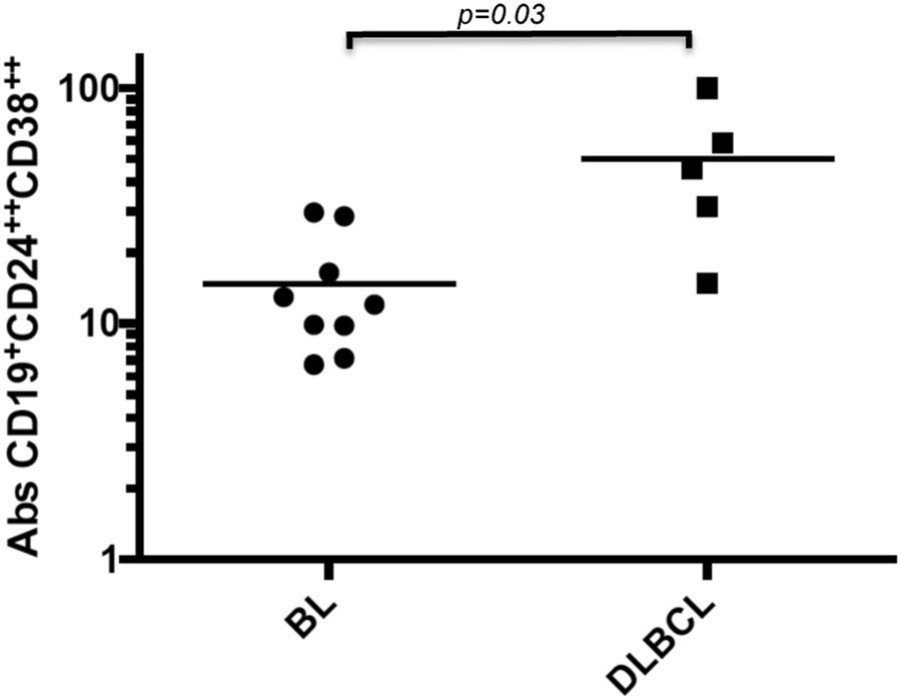
Levels of CD19^+^CD24^++^CD38^++^ B cells were higher preceding DLCL than BL. Multicolor flow cytometry was performed in prospectively collected PBMC from DLBCL and BL 1–4 years prior to AIDS-NHL diagnosis. Absolute numbers of CD19^+^CD24^++^CD38^++^ cells (cells/mm^3^) were measured. p-values were calculated using either an F-test for the difference in log means in a linear mixed model, respectively.

We observed that Breg cells were significantly more elevated 1–4 years before diganosis in those HIV+ subjects who went on to develop AIDS-NHL of the DLBCL subtype, when compared to subjects who developed AIDS-NHL of the BL subtype (p = 0.003) (Fig. 3). In contrast, we did not see any significant difference in the numbers of CD19^+^PD-L1^+^ cells in those HIV+ persons who went on to develop DLBCL or BL (data not shown).

### CD19^+^PD-L1^+^ B cells appear to be a subpopulation of Breg cells

**Figure 4:**
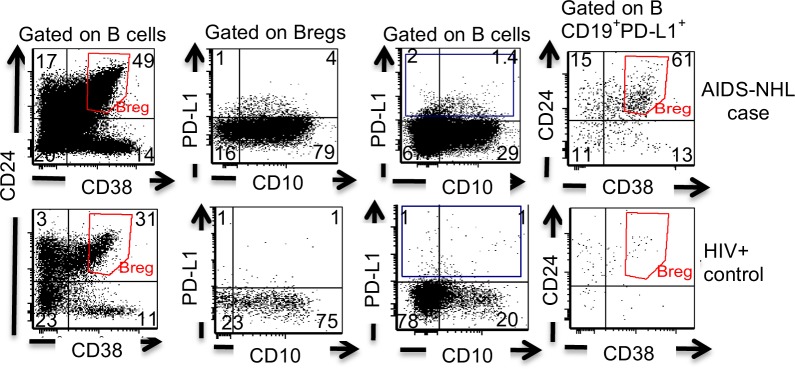
CD19^+^PD-L1^+^ cells are a subpopulation within Bregs in peripheral blood precceding AIDS-NHL diagnosis. Detection of levels of Breg cells (CD19^+^CD24^++^CD38^++^) and CD19^+^PD-L1^+^ cells by multicolor flow cytometry from a representative AIDS-NHL case (1–4 years before diagnosis) and its matched HIV+ control.

Others have noted that Breg cells can express PD-L1^30^. Therefore, we further characterized the phenotype of B cells that express PD-L1 prior to AIDS-NHL diagnosis. To do this, we utilized multicolor flow cytometry to further characterize CD19^+^PD-L1^+^ cells. We observed that the majority of the PD-L1^+^ B cells had a phenotype consistent with that of Breg cells (CD19^+^CD24^++^CD38^++^), indicating that these PD-L1+ B cells are a subpopulation of the so-called Breg cell population, at least in these HIV+ subjects who went on to develop AIDS-NHL (Fig. 4).

### CD40L-containing HIV induces PD-L1 on B cells

**Figure 5:**
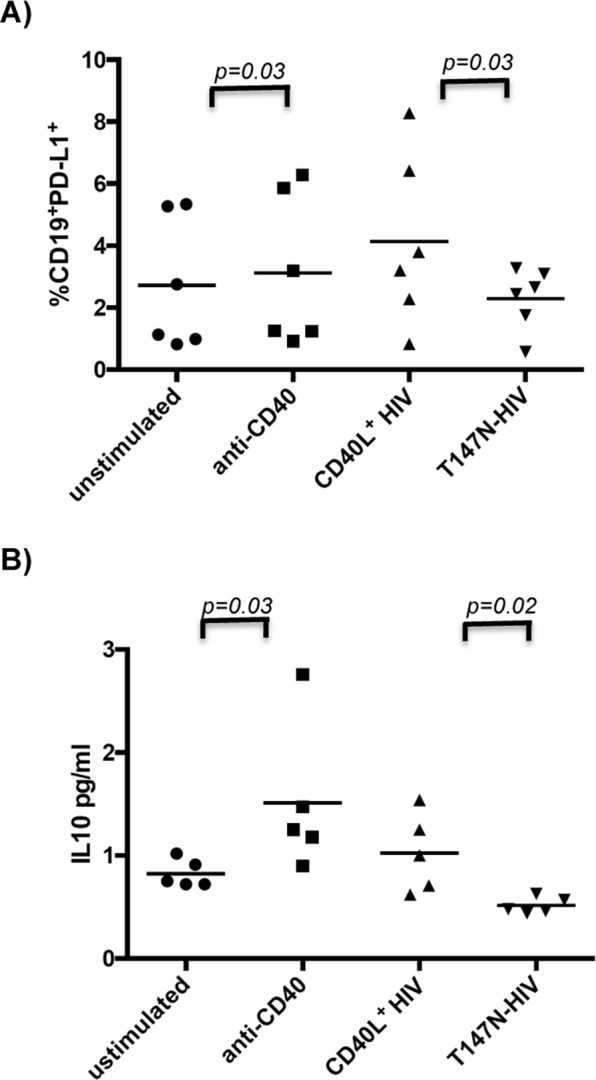
CD40L+ HIV induces PD-L1 expression on B cells, as well as IL10 secretion. Isolated B cells were exposed to mock supernatants, anti-CD40 (positive control) CD40L+ HIV and T147N-HIV (CD40L mutant-HIV) for 2 hours and then cultured at 37 °C for 3 days. (**A**) flow cytometry for PD-L1 was performed on cultured B cells and % of PD-L1+ B cells are shown, (**B**) IL10 levels in supernatants of cultured cells, measured using Luminex multiplexed assays. p-values were calculated using a Wilcoxon test.

CD40 activation is known to lead to Breg cell development^25^. In prior work, we^23,24^ and others^23,24^ found that HIV virions, produced in human T cells, contain CD40L, presumably obtained after lytic infection of these infected host cells. Therefore, we determined whether exposure of B cells to HIV virions up-regulated PD-L1 expression. We stimulated B cells isolated from the peripheral blood of healthy donors with HIV virions that express CD40L on their surface (CD40L+ HIV virions), finding that PD-L1 expression was increased significantly (p = 0.032). In contrast, B cells that were exposed to HIV virions that expressed a non-functional, mutant form of CD40L (T147N-HIV) were not stimulated to express PD-L1 (Fig. 5A). Therefore, CD40L+ HIV virions can directly induce PD-L1 expression on B cells, providing a mechanism for how PD-L1 expression may be driven on these cells by HIV infection. We also measured levels of IL10 in the supernatants collected from B cells exposed to CD40L+ HIV. B cells stimulated with CD40L+ HIV virions were seen to secrete IL10, in addition to expressing PD-L1, indicating that these B cells have the potential to function as Breg cells via both IL10 secretion and PD-L1 expression (Fig. 5B).

## Discussion

Chronic B-cell activation is believed to play an important role in HIV infection-associated immune dysfunction, as well as in the development of AIDS-NHL. However, the mechanisms by which B-cell activation and dysfunction contribute to AIDS-NHL have not been fully defined. Here we show that Breg cells (CD19^+^CD24^++^CD38^++^), as well as B cells expressing PD-L1 (CD19^+^PD-L1^+^ cells), are elevated prior to AIDS-NHL diagnosis. Additionally, we note significant overlap in these B cell subsets, as many Bregs were seen to also express PD-L1. These B cell subsets have the potential to play an important role in lymphomagenesis in a dual fashion, by enhancing B-cell activation (through IL10), and by impairing/inhibiting T cell function (through IL10 and PD-L1), including that of cytotoxic T cells (CTL), which may be involved in the immunoregulation of EBV infected B cells and/or HIV-infected CD4 T cells.

It was previously shown by Siewe *et al*. that Breg cells are elevated prior to AIDS-NHL diagnosis^31^. However this initial study was limited by the small sample size (n = 12) and the proximity of the single sample collection to AIDS-NHL diagnosis (8 months prior to AIDS-NHL). In this larger study, we confirm that Breg cells are significantly elevated in HIV infection, and are even more elevated prior to AIDS-NHL. We also quantified these Breg subsets over a longer time period preceding NHL diagnosis, as well as at two time periods preceding NHL diagnosis. We found that Breg cells were elevated prior to AIDS-NHL diagnosis and observed that Breg cells were elevated up to, but not beyond, 4 years prior to AIDS-NHL, when compared to HIV+ controls. However, Breg cells were significantly elevated 1–4 years prior to AIDS-NHL diagnosis when compared to Bregs from more than 4 years prior to AIDS-NHL diagnosis in the same individual, suggesting that levels of Bregs increase approaching AIDS-NHL diagnosis. These observations suggest that elevated numbers of Breg cells in the peripheral blood of HIV+ subjects is a characteristic of subjects who develop AIDS-NHL, and that Breg cells may be involved in the pathogenesis of these cancers. We also observed significantly higher numbers of Breg cells in those who developed DLBCL, compared to those who developed BL. However this finding is tempered by the relatively low number of cases studied, due to stratification by AIDS-NHL subtype.

It has been shown that Breg cells from HIV+ subjects are able to inhibit CTL HIV-specific responses in a dual fashion, by secreting the T cell inhibitory cytokine IL10 and via ligation of PD1 on T cells by PD-L1 on B cells^32^. Here we show, as others have recently shown^33^, that higher numbers of B cells expressing PD-L1 are present in the peripheral blood of HIV infected individuals. Additionally, we show that HIV+ subjects who went on to develop AIDS-NHL have even higher numbers of CD19^+^PD-L1^+^ B cells, when compared to HIV+ controls in peripheral blood. PD-L1 is an immunomodulator, and is of great importance, since exhausted non-functional T cells express PD1 in HIV infection. The higher expression of PD-L1 on B cells in HIV infection raises the possibility that these CD19^+^PD-L1^+^ B cells may be interacting with T cells through PD1, contributing to the development of AIDS-NHL by inhibiting/impairing CD4^+^ and CD8^+^ T cells, which play an important role in dampening the growth of EBV-infected B cells and which can effect anti-tumor responses (Fig. 5).

Additionally, we show that exposure to HIV virions expressing CD40 ligand can modestly induce the expression of PD-L1 on B cells, as well as IL10 secretion. Lopez-Avente *et al*. recently showed that HIV can directly induce a regulatory B cell-like immunosuppressive phenotype (CD19^+^CD24^++^CD38^++^)^34^. Here, we extend their observations by showing that HIV may be inducing PD-L1 in this Breg cell phenotype, at least in part, through the expression of CD40L on HIV virions, which can interact with CD40 on B cells. This provides one mechanism by which PD-L1^+^ Breg cells may arise in HIV infection. It is important to note that AIDS-NHL tumor cells express PD-L1^35^. The expression of PD-L1 on tumor cells can allow such cells to evade immune surveillance^36^. Together, these findings lead us to speculate that the induction of PD-L1 on B cells may be an early event driving lymphomagenesis. Certainly, more work needs to be done to elucidate the role of PD-L1^+^ B cells in lymphomagenesis.

## Methods

### Study design and population

The study design is a nested case-control study within the Multicenter AIDS Cohort Study (MACS) cohort. The MACS is a prospective cohort study of the natural and treated history of HIV infection and AIDS^37^. We obtained 35 viable frozen peripheral blood mononuclear cells (PBMC) from individuals who developed NHL, collected prior to their NHL diagnosis (cases), from the UCLA MACS repository. We studied samples from the same individual collected at two different MACS study visits: one at >4 years, and another at 1–4 years prior to NHL diagnosis (n = 18). For inidividuals (n = 11) who did not have two serial samples, we used the one sample that was available in the analysis. We also obtained similar samples from 29 HIV+ patients who did not develop NHL (HIV+ controls), matched to cases by date of birth (within 200 days), study visit (within 250 days) and CD4 count (±175cells/mm^3^), plus samples from an additional 15 HIV-negative controls obtained from the MACS, matched on study visit. The MACS was approved by the human subjects research review committees at all participating institutions; all human subjects participating in the MACS provided written informed consent. The specimens and clinical information provided by the MACS were stripped of any personal identifying information.

### Flow cytometry

Multicolor flow cytometry was performed on 1 × 10^6^ PBMC, for the following immune markers: CD3, CD4, CD19, CD24, CD38, CD71, CD1d, PD-L1 (eBioscience) and CD10 (Becton Dickenson, BD). These antibodies were conjugated with FITC, PE, APC, PerCp-Cy5.5, PE-Cy7, APC-Alexa750, Pacific blue, 605eFluor or 650 eFluor (nano-crystals), respectively. Stained samples were run in a LSR Fortessa.

### Statistics

Log-transformed means were compared between NHL HIV+ individuals and HIV+ controls using linear mixed models with a random effect for each pair examining each visit (>4 years and 1–4 years prior to NHL diagnosis) independently and also combined. When visits were combined in the same analysis, we evaluated the change in values across visits via an interaction term comprising an additional random effect for each individual to account for multiple observations per individual. We also compared log-transformed means between HIV+ controls and HIV-negative controls using linear regression models controlling for age. P-values were obtained from a t-test for the difference in corresponding means. When appropriate, least-square means are presented. Analyses were done using the R statistical language package. p-values were also calculated using a Wilcoxon test.

### B-cell exposure to HIV

B cells were isolated from fresh PBMC, obtained from HIV uninfected donors, by negative selection using the RosetteSep Human B Cell Enrichment kit (Stem Cell Technologies). B cells were exposed to X4-HIV strain (NL4–3) and/or an R5-HIV strain (JR-CSF) virions, containing or not containing CD40L, or T147N (non-functional CD40 mutant), at a concentration of 100 ng p24 per 1 × 10^6^ B cells, or to anti-CD40 agonistic antibody (1 μg/ml) as a positive control (Biolegend), incubated for 2 hours, then plated at a concentration of 0.5 × 10^6^ cells per ml, left at 37 °C for 3 days^23^, and stained as described above^23^. Supernatants of these cultures were assessed using Luminex-based high-sensitivity multiplexed immunometric assays (R&D Systems) to determine human cytokine levels. This assay can simultaneously measure levels of the following human cytokines: GM-CSF, IFNγ, IL1β, IL2, IL4, IL5, IL6, IL7, IL8, IL10, IL2 (p70), IL13 and TNFα^23^.

### Ethics statement

This study involved the use of samples obtained from human subjects. Multicenter AIDS Cohort Study (MACS) at UCLA, which provided specimens and data obtained from human subjects, with personal identifying information removed, was approved by the Institutional Review Board (IRB) of the UCLAof the UCLA Human Research Protection Program (HRPP). All participants in the MACS provided written informed consent. The current study was determined by the UCLA IRB to be exempt from IRB review, as the information was provided in such a manner that subjects cannot be identified, directly or through identifiers linked to the subjects

## Data Availability

The datasets used and/or analyzed during the current study are available from the corresponding author on reasonable request. Methods were performed in accordance to the relevant guidelines and regulation.
