# Author Correction: Elevated numbers of PD-L1 expressing B cells are associated with the development of AIDS-NHL

**DOI:** 10.1038/s41598-020-57442-8

**Published:** 2020-01-15

**Authors:** Marta Epeldegui, David V. Conti, Yu Guo, Wendy Cozen, Manuel L. Penichet, Otoniel Martínez-Maza

**Affiliations:** 1 UCLA AIDS Institute, University of California, Los Angeles, California USA; 2 Jonsson Comprehensive Cancer Center, University of California, Los Angeles, California USA; 3 Department of Obstetrics and Gynecology, David Geffen School of Medicine, University of California, Los Angeles, California USA; 4 Department of Preventive Medicine Keck School of Medicine and Norris Comprehensive Cancer Center, University of Southern California, Los Angeles, California USA; 5 Department of Pathology, Keck School of Medicine and Norris Comprehensive Cancer Center, University of Southern California, Los Angeles, California USA; 6 Division of Surgical Oncology, Department of Surgery, David Geffen School of Medicine, University of California, Los Angeles, California USA; 7 Department of Microbiology, Immunology, and Molecular Genetics, David Geffen School of Medicine, University of California, Los Angeles, California USA; 8 The Molecular Biology Institute, University of California, Los Angeles, California USA; 9 Department of Epidemiology, UCLA Fielding School of Public Health, University of California, Los Angeles, California USA

Correction to: *Scientific Reports* 10.1038/s41598-019-45479-3, published online 28 June 2019

This Article contains an error in Figure 2. Panels 2B and 2C were published as Figures 2C and 2B respectively. The correct Figure 2 appears below as Figure 1.

**Figure 1:**
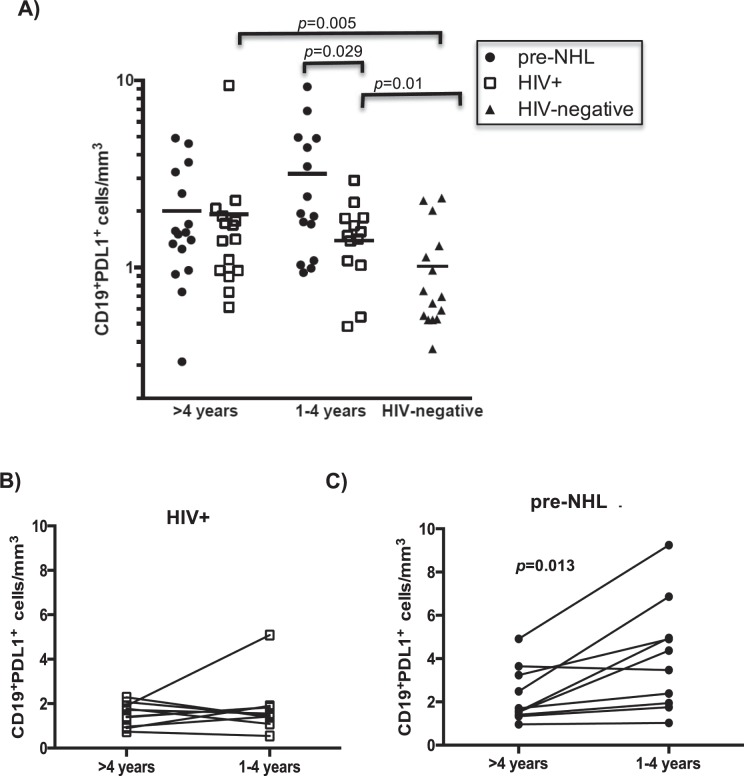
.

